# Modeling the Calvin-Benson cycle

**DOI:** 10.1186/1752-0509-5-185

**Published:** 2011-11-03

**Authors:** Jiri Jablonsky, Hermann Bauwe, Olaf Wolkenhauer

**Affiliations:** 1 Department of Systems Biology and Bioinformatics, University of Rostock, 18051 Rostock, Germany; 2 Department of Plant Physiology, University of Rostock, 18059 Rostock, Germany

## Abstract

**Background:**

Modeling the Calvin-Benson cycle has a history in the field of theoretical biology. Anyone who intends to model this system will look at existing models to adapt, refine and improve them. With the goal to study the regulation of carbon metabolism, we investigated a broad range of relevant models for their suitability to provide the basis for further modeling efforts. Beyond a critical analysis of existing models, we furthermore investigated the question how adjacent metabolic pathways, for instance photorespiration, can be integrated in such models.

**Results:**

Our analysis reveals serious problems with a range of models that are publicly available and widely used. The problems include the irreproducibility of the published results or significant differences between the equations in the published description of the model and model itself in the supplementary material. In addition to and based on the discussion of existing models, we furthermore analyzed approaches in PGA sink implementation and confirmed a weak relationship between the level of its regulation and efficiency of PGA export, in contrast to significant changes in the content of metabolic pool within the Calvin-Benson cycle.

**Conclusions:**

In our study we show that the existing models that have been investigated are not suitable for reuse without substantial modifications. We furthermore show that the minor adjacent pathways of the carbon metabolism, neglected in all kinetic models of Calvin-Benson cycle, cannot be substituted without consequences in the mass production dynamics. We further show that photorespiration or at least its first step (O_2 _fixation) has to be implemented in the model if this model is aimed for analyses out of the steady state.

## Background

**Figure 1:**
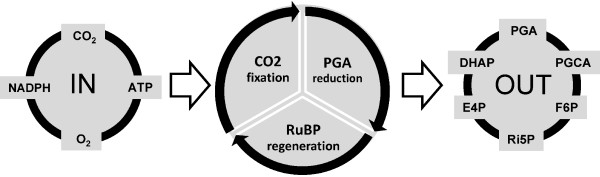
**Scheme of inputs, three main phases and outputs of the Calvin-Benson cycle in cyanobacteria**. The initial phase of the Calvin-Benson cycle is fixation of CO_2 _into carbon skeleton - carboxylation of ribulose-1,5-bisphosphate (RuBP). The second phase describes the reduction of the 3-phosphoglycerate (PGA) which forms the glyceraldehyde phosphate and dihydroxyacetone phosphate. The final regeneration phase of the cycle involves several reactions leading to the RuBP reassembling.

The Calvin-Benson cycle is a central part of the carbon metabolism in oxygenic photosynthesis, involving 11 different enzymes that catalyze 13 reactions [1]. The cycle is an open system, connected to light photosynthetic reactions, CO_2 _fixation and other parts of carbon metabolism (Figure 1), e.g., starch and sucrose synthesis. It is this complexity that motivates the use of mathematical modeling to unravel the dynamic regulation that underlies experimental observations of the Calvin-Benson cycle.

There are two common approaches used in modeling the Calvin-Benson cycle: kinetic modeling, e.g., [2,3] and stoichiometric modeling, e.g., [4-6]. Kinetic modeling requires to obtain/have available kinetic properties of the enzymes involved. These are mostly known if one assumes conservative kinetic parameters among the species with several different ways of CO_2 _fixation. On the other hand, stoichiometric modeling allows an analysis of the entire carbon metabolism within the given constraints and without the need of kinetic properties. It would seem plausible to combine both approaches and overlap fluxes from the kinetic model of the Calvin-Benson cycle with the stoichiometric analysis of the metabolic network [7,8].

Even though it is a core part in a variety of photosynthetic models or projects modeling the entire photosynthesis [9,10], kinetic modeling has mostly focused on the stability of the Calvin-Benson cycle itself and continues to attract considerable scientific interest [11,12]. To anyone entering this field, it is clear that the Calvin-Benson cycle is the best-studied plant metabolic system. Since modeling the Calvin-Benson cycle has a long history, the question arises how one should proceed with a further analysis of this system. The standard scientific approach is to build on existing knowledge and models. A natural progression would thus to adapt existing models, to refine and expand them for adjacent metabolic pathways.

What our present work reveals is that, without significant modifications and corrections of the well-established models, it is not possible to re-use existing models. Our analysis shows that one of the existing and widely used models produces an "accumulation" of starch in high *negative *concentration with consequences to the behavior of the whole system (apart from being biologically implausible). In another case an interchanged parameter leads to 100-times faster equilibrium in one particular reaction, which significantly shifted the steady states of all metabolites. We furthermore found errors, which have propagated from one generation (publication) to another. Our study highlights difficulties with the reproducibility of model-based results, provides solutions and suggestions on how to proceed with extensions to existing models of the Calvin-Benson cycle.

In order to support the evolution of models for the Calvin-Benson cycle and to improve the reproducibility of model-driven results, we discuss ways to improve and correct existing models. We also open a discussion about how to connect the Calvin-Benson cycle with other parts of the carbon metabolism in the case of cyanobacteria and in relation to the system's efficiency, regulation, mass production and implementation of photorespiration.

## Methods

To investigate mathematical and computational models for their suitability and possible errors all models were encoded both in Matlab, using the SimBiology toolbox (MathWorks Inc., Natick, Massachusetts, USA) and at the same time in Copasi [13]. For transparency and to allow readers to use corrected models of the Calvin-Benson cycle, we make all files and data required for the simulation publicly available in additional files (.xml).

## Results and Discussion

### Available kinetic models for the Calvin-Benson cycle

There are studies that successfully use a simplified description of the Calvin-Benson cycle, for instance the photosynthetic oscillations in the chlorophyll fluorescence [14]. However, if one analyses the regulation of carbon metabolism, such study cannot be done without a detailed kinetic model of the Calvin-Benson cycle. Since kinetic modeling of the Calvin-Benson cycle has a long tradition [2,3,15-22], the standard approach would be to adapt, refine and expand an existing model. Focusing on the Calvin-Benson cycle and carbon metabolism, we have investigated several well-established models of this system, which are discussed in further detail below.

### Hahn models

This fundamental model was presented by Hahn [15] who also published further related models of the Calvin-Benson cycle [16,17]. The kinetic parameters in all models were chosen on the basis of a realistic photosynthetic rate in order to give reasonable steady-state values for metabolites. One would assume that the earlier model [15] was extended by photorespiration in the next model generation [17]; together with the realistic response to the changes in the O_2 _and CO_2 _concentrations [17]. This assumption is based on the fact that the equations describing the Calvin-Benson cycle are the same in both models [15,17]; however, some values of the kinetic parameters differ significantly.

The most prominent example is the rate of the CO_2 _fixation expressed by the rate constant k_1_. The value of this calculated parameter is in both models identical [15,17], i.e. on the basis of estimated gross photosynthetic rate per unit area of leaf tissue $\left(P_{g}^{A}\right)$. Since the $\left(P_{g}^{A}\right)$ is the same in both models, it is not clear why value of k_1 _is 57.3 - fold higher in the later model [17]. Hahn mentions that the value of k_1 _was, in order to compensate the neglected photorespiration in the earlier model [15], decreased (without any detail regarding the original value of k_1_). However, it is unlikely that the original and unknown value of k_1 _in the earlier model [15] is 57.3 - fold lower due to photorespiration because the author himself assumed in the later model [17] the ratio for the carboxylase/oxygenase activity of RuBisCO (Ribulose-1,5-bisphosphate carboxylase oxygenase) equal to 3.7.

Nevertheless, we have encoded the earlier Hahn model [15] into biochemical simulators according its original description. Having only the rate constants, we employed mass action kinetics as the author did [15]. For initial concentrations we used the steady-state values calculated by Hahn [15].

**Figure 2:**
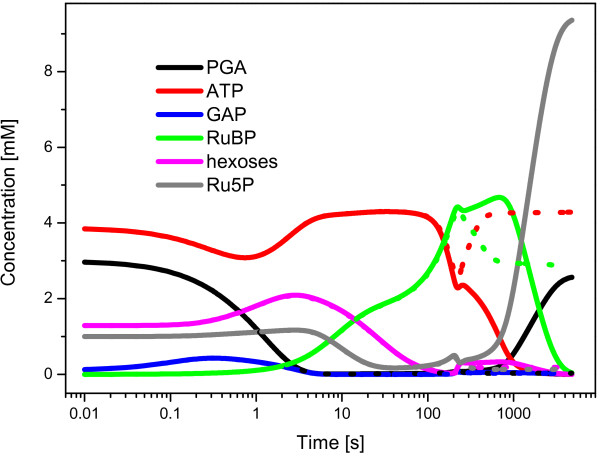
**Stability test based on the Hahn model**. The stability test was performed in the same conditions as author did (Figure 2 in [15]): all state variables were set to their calculated steady-state values, except RuBP (ribulose-1,5-bisphosphate) which was set to zero. Dotted lines stand for the scenario with fixed (i.e., constant) concentration for external phosphate during the simulation. Stability test performed by author in the original study [15] ran only 80 s, our test lasted 80 minutes.

In a pilot simulation we have encountered the very low level of ATP in the steady-state; ATP **· **(ADP + ATP)^-1 ^= 0.03. We have therefore performed a stability test in the same condition as author did (Figure 2 in [15]), i.e., all state variables were set to their calculated steady-state values, except RuBP (ribulose-1,5-bisphosphate) which was set to zero, simulation ran 80 minutes instead of 80 s in the original work. One can draw two major conclusions from the results of the stability test, which is shown in Figure 2. At first, the phosphorylation of PGA to BPGA (glycerate-1,3-bisphosphate) is much faster than the rate of CO_2 _fixation; moreover the CO_2 _fixation is too slow, such that we can even observe a gradual accumulation of RuBP. Secondly, the ATP level is dropping down in 88 s of the simulation which leads to an accumulation of Ru5P (ribulose-5-phosphate) and consequent suspension of the cycle. We note that the stability test performed by the author lasted only 80 s.

The reason for the drop in the ATP level in the certain time point (Figure 2) was a depletion of both intra- and extracellular phosphates (data not shown). Finally, we achieved a stable solution by fixing (having constant concentration) the Pi_ext (extracellular phosphate). This approach prevents the suspension of the cycle (Figure 2) but the steady-state of the system is still far away from known values (e.g., the aforementioned range for PGA) or results presented by author (Figure 2 in [15]).

### Pettersson model

**Figure 3:**
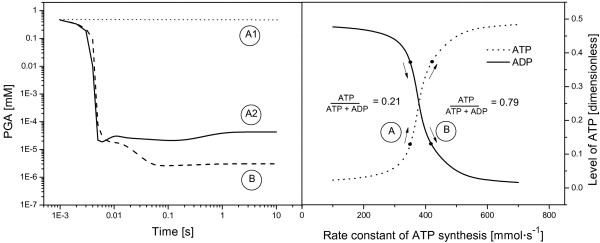
**Analysis of the steady-state concentration of PGA in dependence of ATP level based on the Pettersson model**. **A**: original setting for ATP synthesis and corresponding steady-state concentration of PGA. **A1**: expected value based on the original publication [2]. **A2**: value from our simulation based on rewritten Pettersson model. **B**: our modification employing the physiological level of ATP and corresponding steady-state concentration of PGA.

The Pettersson model [2] reflects a significant effort in the elaboration of the kinetic behavior and control properties of the Calvin-Benson cycle. Our analysis of this model revealed two problems: an insufficient rate of the ATP synthesis (Figure 3A) and we did not reach the same results as the authors did (published data for extracellular phosphate Pi_ext = 0.5 mM). For instance, the steady state concentration for PGA is 13 817 - fold lower (i.e., 42.7 nM) in comparison to the calculated value 0.59 mM reported in the original publication [2], see Figure 3A1-A2. Finally, if we move to the physiological range for the ATP concentration given by ATP · (ADP + ATP)^-1 ^ratio [23], concentration of PGA further decreases (3.03 nM, Figure 3B). This shift indicates that the chosen set of kinetic parameters in the Pettersson model does not describe the reality in which the steady-state concentration of PGA was reported to be in the range 2.15 mM [24] - 11.7 mM [25].

### Poolman and Zhu-Laisk models

Although it is not a problem to reconstruct the established fundamental models from their description, a problem is that our simulations based on the Hahn and Pettersson models demonstrate totally different behaviors in comparison to what was presented by their authors [2,15]. It is not clear what the problem is because the computational methodology used for running the simulations has not been described in the publications. But if one employs well established computational methods and tools (e.g., Matlab), the original results [2,15] are not reproducible. Therefore, if one wants to study the cycle itself or expand the model by adding other part(s) of the carbon metabolism, the reasonable cause of action would be to look for available models for which one knows all details about the computational methods. Such models can be nowadays found in model databases (e.g., http://www.ebi.ac.uk/biomodels-main) or they are made available as a supplement to a publication. For the Calvin-Benson cycle, there are two main options, the Poolman model [21] and the Zhu model [3].

The Poolman model is available as a .xml file (http://www.ebi.ac.uk/biomodels-main/) and can be therefore easily imported to any biochemical simulator supporting SBML (system biology markup language) level 2. We have employed both Copasi [13] and the SimBiology toolbox for Matlab (MathWorks) to study this model. The Poolman model is structurally very close to the Pettersson model [2] but with an altered kinetic parameters. One can speculate that the author encountered the same problems with Pettersson model as we have and as a result, employed different set of kinetic parameters. This model was used as an example to illustrate possible applications in the metabolic modeling [21] and stability analysis (e.g., number of steady states) rather [22] than for kinetic modeling of the particular metabolite(s). The initial conditions were exactly the same as in the original work [21].

Pilot simulations based on the Poolman model showed that the system reached the steady state at 0.6 s from the start of the simulation. If the initial concentration of PGA (3-phosphoglycerate) is increased 10-times, the steady state is reached after 1.4 s. This feature of the model is based on the assumption that metabolites in the system are maintained at a fast equilibrium [2]. Furthermore, to ensure the assumed fast equilibrium, rate constants in the Poolman model were set to extremely high values. For example, the rate constants for reactions catalyzed by transketolase (equations E7 and E10, [21]) are equal to 500 000 000 l·mmol ^-1^·s^-1 ^[21] and the flux through these reactions is 39.7 mmol·s^-1 ^(our calculation based on the unmodified Poolman model [21]). This flux is in sharp contrast to the proposed (0.1 mmol·s^-1 ^[19]) and measured (0.36 mmol·s^-1 ^[26]) maximal rates for these reactions.

The Poolman model reaches its equilibrium at 0.6 s of the simulation also in other cases (after dark → light switch or changes in CO_2 _level). On the other hand, it is known that the real system is limited by RuBisCO (ribulose-1,5-bisphosphate carboxylase/oxygenase) activation whose induction phase takes 120 s [27] - 600 s [28]. We note that the activation of RuBisCO was also considered in a kinetic model with the time constant equals to 250 s [14].

The Calvin-Benson cycle is an open system with many inputs and outputs (Figure 1). It is therefore important to analyze its output behavior as well. To that end, we have changed the settings for sinks and starch in the Poolman model from the fixed (constant) concentration to variable concentration (responding according the reactions and differential equations). We note that the original setting for other fixed metabolites, i.e., for inputs (CO_2_, cytosolic Pi, NADPH, NADP^+ ^and H^+^) was not changed.

**Figure 4:**
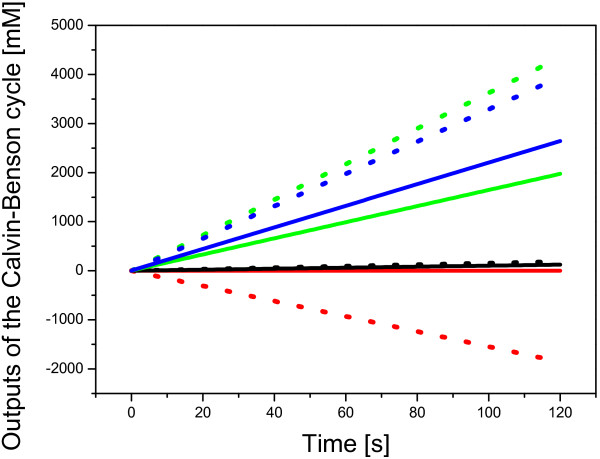
**Analysis of the outputs from the Calvin-Benson cycle based on the Poolman model**. This analysis shows the difference between the original Poolman model [21] and the revised version in which the rate equation E17 has been modified (replacement of Pi_ch by starch). This modification solved the problem with negative concentration of the starch. **Dotted line **stands for the original Poolman model, **solid line **for the modification/correction. Meaning of the colors: **red **- starch, **black **- GAP in cytosol, **green **- PGA in cytosol, **blue **- DHAP in cytosol.

Analysis of sinks showed an expected accumulation of the cytosolic GAP (glyceraldehyde 3-phosphate), DHAP (dihydroxyacetone-phosphate) and PGA, see Figure 4, but the system multiplied its initial metabolites pool 61-fold at 10 s of the simulation. More strikingly, allowing changes in the starch concentration revealed that the starch is not only degraded but reached a high negative concentration, -14.1-fold of the initial pool of metabolites at 10 s of the simulation, as shown in Figure 4. The negative flux through the starch degradation pathway increased the rate of sinks accumulation, i.e., the mass production, by 40% (Figure 4). It was caused by error in the equation describing the starch degradation (equation E17 in the original work [21]). This problem with negative starch concentration can be either bypassed by switching off this reaction, which would set the system for the slow starch synthesis, or solved (Figure 4) by a replacement of Pi_ch by starch in the original [21] rate equation:

$$\frac{\text{StPase}\_\text{VM}\times Pi\_ch}{Pi\_ch+\text{StPase\_km}\times (1+\frac{\text{G}1\text{P\_ch}}{\text{StPase\_kiG}1\text{P}}}$$

The Poolman model was designed for other purposes (stability analysis) but it is clear that this model cannot be used for the purpose of comparison with experimental time-series data without changing both the equations and kinetic parameters.

The remaining option is the Zhu model [3]. Zhu and coworkers presented a model of C_3 _photosynthesis, extending the Laisk model [19] by addition of the photorespiratory pathway. Since we do not focus on the complete photorespiratory pathway in this study, we did not analyze the Laisk model separately, except for the difference in the case of phosphate translocator (see the section *Calvin-Benson cycle as a part of the metabolic network: PGA sink implementation*).

The Zhu model is a natural starting point and suitable template for studying photorespiration, even if it is not easily accessible and understandable because it is encoded in 34 Matlab files. The metabolites in the Zhu model reach the equilibrium at 150 s of the simulation (data not shown), which is in agreement with previous findings [27] and in contrast to 0.6 s based on the Poolman model [21]. However, several discrepancies and serious errors in the Zhu model, particularly in the part describing the Calvin-Benson cycle, can be found. The discrepancies in the Zhu model include interchanged values of k_E13 _and k_I135 _in Appendix C and the nowhere used constant K_M8_, make the description of the model slightly confusing. The following section is dedicated to the analysis of the errors occurring in the Zhu model, as well as necessary modifications for this model.

### Analysis of the Zhu model and problems occurring in kinetic modeling of the Calvin-Benson cycle

The programming environments used for kinetic modeling of the Calvin-Benson cycle include Turbo Pascal, used in [19]; Scampi, used in [21] or Matlab, used in [3]. Command-line programming is more prone to errors and also makes it more likely for errors to propagate into new models. For example, in the Laisk model [19] one error propagated into other models due the missing dimensional analysis in Pascal. This problem can also be found in the last generation of the model containing the Calvin-Benson cycle, in the Zhu model [3]. The following problems occur in the Zhu model:

• Two very different descriptions for reactions v7: S7P + GAP = Ri5P + Xu5P and v10: F6P + GAP = E4P + Xu5P), one in the paper (Appendix A) and another one encoded in the MATLAB file PSRate.m in supplement of the paper [3].

• Wrong dimension for equations v7 and v10.

• Kinetic parameters were incorrectly taken from kinetic characterization of transaldolase instead of transketolase.

• The value of the equilibrium constant k_E7 _in equation v7 is 100 times higher than what is known from literature [1,19].

In order to analyze these problems, we have rewritten the 2007 Zhu model, originally encoded in Matlab, for use with Copasi [13] and also rewrote it for the SimBiology. Since all errors occur in the description of the Calvin-Benson cycle, the photorespiratory pathway was not considered in the rewritten Zhu model. Finally, we improved the Zhu model in a way that all metabolites have their own concentrations, e.g., we consider separated DHAP and GAP instead of T3P pool, in comparison to original 2007 Zhu model. We then compared the steady-state values of the metabolites in dependence of ATP · (ADP + ATP)^-1 ^ratio, reflecting the energy limitation [23]. Since all errors are related to the transketolase, we discuss the consequence only for two substrates of transketolase, F6P (fructose 6-phosphate) and S7P (sedoheptulose-7-phosphate).

**Figure 5:**
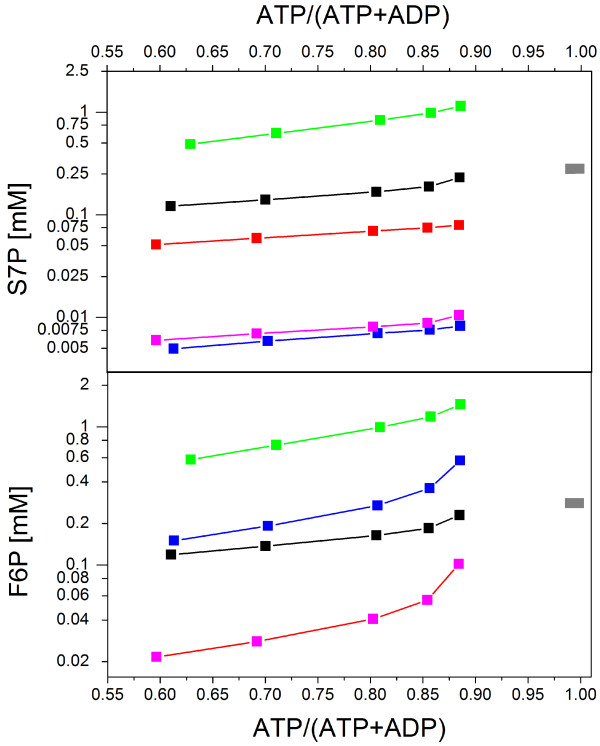
**Analysis of the steady state values for S7P and F6P in dependence of ATP concentration**. This analysis is based on the comparison of original [3] and corrected Zhu model. Furthermore, an impact of particular problems is analyzed as well. The modifications in kinetic parameters and the rate equations are described by different colors. **Black**: original Zhu model, Appendix A version; **red**: original Zhu model, Matlab version [3]; **gray**: modified Appendix A version [3] (k_M103 _= 0.015 mmol·l^-1^); **violet**: modified Matlab version (k_M103 _= 0.015 mmol·l^-1^); **blue**: modified Matlab version (k_M103 _= 0.015 mmol·l^-1^, k_E7 _= 0,076) and **green**: modified Appendix A version (k_M103 _= 0.06 mmol·l^-1^, k_E7 _= 0,076). The initial concentrations and conditions were the same for all simulations.

At first, we analyzed the key problem of the Zhu model - that there are actually two versions of this model due to the different descriptions of reactions catalyzed by transketolase, i.e., equations v7 and v10. The first version of the Zhu model can be found in Appendix A [3] (based on the Laisk model [19]) and the other one is in the supplemental Matlab files [3]. It is not clear which encoding was actually used for simulations in the main part of the published paper. If the equations from Appendix A are employed, the steady-state concentrations of F6P and S7P are 3.3-fold and 2.5-fold higher, respectively, in comparison to the model encoded in Matlab, as indicated in Figure 5 (black and red lines).

The case with two model versions gets more complicated because both of them suffer from other problems. The first one, related only to the version in Appendix A [3], is the consequence of a wrong dimension for equations describing the reactions catalyzed by transketolase (v7 and v10) and has its origin in the Laisk model [19]. The problem is that the modifiers E4P (erythrose 4-phosphate) and Ri5P (ribose 5-phosphate) were multiplied instead of added in one of the equations (TEMP1, [3]) employed in the aforementioned reactions. Even if this particular error has an negligible impact in the calculation itself (< ± 1%, data not shown), the incorrect rate equations do not allow a computation of the simulations in biochemical simulators that test the dimension of equations (e.g., SimBiology toolbox).

An alternative but not plausible approach was employed in the case of kinetic parameters for rate equation v10 in the Zhu model. Zhu and co-workers employed different kinetic parameters [29] in comparison to Laisk model [19]. The reason why this is not justified is that the new kinetic parameters were taken from transaldolase [29] instead of transketolase. The fact that the source of kinetic parameters is the non-photosynthetic organism *Dictyostelium discoideum *might be justified. However, we cannot overlook the fact that the transaldolase catalyses the same reaction as transketolase but in opposite direction; it is localized in the pentose phosphate pathway.

One question that arises is why the original kinetic parameters for the rate equation v10 from the Laisk model [19] were not employed in the Zhu model. Strikingly, the original parameters from the Laisk model [19] block the cycle and lead to an accumulation of F6P and S7P, see Figure 5 (gray). This behavior is caused primarily by big difference between the original [19] and new [29] value of the parameter k_M103_, 0.015 mmol·l^-1 ^and 0.46 mmol·l^-1^, respectively. In order to sustain a stable solution, the value of the parameter k_M103 _in the rate equation v10 must be equal or higher than 0.06 mmol·l^-1 ^(data not shown). This problem occurs, however, only for model version from Appendix A [3],. The Matlab model version [3] can employ the original parameter k_M103 _= 0.015 mmol·l^-1 ^from Laisk model [19] and sustains the stable solution sensitive to the changes of ATP level, see Figure 5 (violet).

Finally, and probably the most visible error in the Zhu model is an incorrect value for the equilibrium constants k_E7_, that is, 10 [3] instead of 0.076 [1] or 0.1 [19]. The consequence is a theoretical 100-fold increase in the forward rate of the reaction F6P + GAP = E4P + Xu5P, which is able to shift the equilibrium of entire model and reduce the starch synthesis. In order to analyze the possible impact, we performed the simulations based on both Appendix A (with k_M103 _= 0.06 mmol·l^-1^) and the Matlab version of Zhu model, both with the correct value of k_E7 _= 0.076 [1]. At first, we can compare original versions of Zhu model after correction on k_E7_. The steady state concentrations of F6P and S7P are now 6- and 4.8-fold higher, respectively, if we compare the original [3] and corrected Appendix A model (additional file 1). This is shown in Figure 5 in black and green. In the case of the original [3] and here corrected Matlab version of the Zhu model (additional file 2), the steady state concentrations of F6P is increased by 6.2-fold but S7P is 9.9-fold decreased, respectively; see Figure 5 - red and blue. The quantitative differences in the behavior between these two versions of the Zhu model suggest that one should really speak about two different models rather than two versions of one model. This conclusion is supported by final comparison of both corrected models - the difference in steady state concentration of S7P between both Zhu models is 121-fold as shown in Figure 5 - green and blue.

### Calvin-Benson cycle as a part of the metabolic network: PGA sink implementation

We have revealed several problems with the commonly cited model of the Calvin-Benson cycle. Having in mind that the Zhu model, and others models as well [19,21], considers also the phosphate translocator, an essential question arises of how the errors or generally any changes in the Calvin-Benson cycle can influence other parts of carbon metabolism. The starting point for our analysis was naturally the Laisk model [19] where one can find the most complex description of the phosphate translocator. The approach employed in Laisk model for the phosphate translocator between stroma of chloroplast and cytosol, is very complex. We therefore focused our effort only on the analysis of the PGA export, PGA_stroma _↔ PGA_cytoso_l, described by equation V_PG_Aout in the Laisk model [19]:

$$\begin{array}{l} \frac{{\text{Vm}}_{11}\text{*}\left(\frac{\text{OPc}}{{\text{Km}}_{\text{OPc}}}+\frac{\text{PGAc}}{{\text{Km}}_{\text{PGAc}}}+\frac{\text{GAPc}+\text{DHAPc}}{{\text{Km}}_{\text{T}3\text{Pc}}}\right)\text{*}\left(\frac{\text{PGA}}{{\text{Km}}_{\text{PGA}}}\right)}{\frac{\text{OP}}{{\text{Km}}_{\text{OP}}}+\frac{\text{PGA}}{{\text{Km}}_{\text{PGA}}}+\frac{\text{GAP}+\text{DHAP}}{{\text{Km}}_{\text{T}3\text{P}}}+\;\frac{\text{OPc}}{{\text{Km}}_{\text{OPc}}}+\frac{\text{PGAc}}{{\text{Km}}_{\text{PGAc}}}+\frac{\text{GAPc}+\text{DHAPc}}{{\text{Km}}_{\text{T}3\text{Pc}}}+\text{ SUBST}}\;\\ -\;\frac{{\text{Vm}}_{11}\text{*}\left(\frac{\text{OP}}{{\text{Km}}_{\text{OP}}}+\frac{\text{PGA}}{{\text{Km}}_{\text{PGA}}}+\frac{\text{GAP}+\text{DHAP}}{{\text{Km}}_{\text{T}3\text{P}}}\right)\text{*}\left(\frac{\text{PGAc}}{{\text{Km}}_{\text{PGAc}}}\right)}{\frac{\text{OP}}{{\text{Km}}_{\text{OP}}}+\frac{\text{PGA}}{{\text{Km}}_{\text{PGA}}}+\frac{\text{GAP}+\text{DHAP}}{{\text{Km}}_{\text{T}3\text{P}}}+\;\frac{\text{OPc}}{{\text{Km}}_{\text{OPc}}}+\frac{\text{PGAc}}{{\text{Km}}_{\text{PGAc}}}+\frac{\text{GAPc}+\text{DHAPc}}{{\text{Km}}_{\text{T}3\text{Pc}}}+\text{ SUBST}}\\ \text{SUBST}=\;\left(\frac{\text{OP}}{{\text{Km}}_{\text{OP}}}+\frac{\text{PGA}}{{\text{Km}}_{\text{PGA}}}+\frac{\text{GAP}+\text{DHAP}}{{\text{Km}}_{\text{T}3\text{P}}}\right)\text{*}\left(\frac{\text{OPc}}{{\text{Km}}_{\text{OPc}}}+\frac{\text{PGAc}}{{\text{Km}}_{\text{PGAc}}}+\frac{\text{GAPc}+\text{DHAPc}}{{\text{Km}}_{\text{T}3\text{Pc}}}\right) \end{array}$$

**Figure 6:**
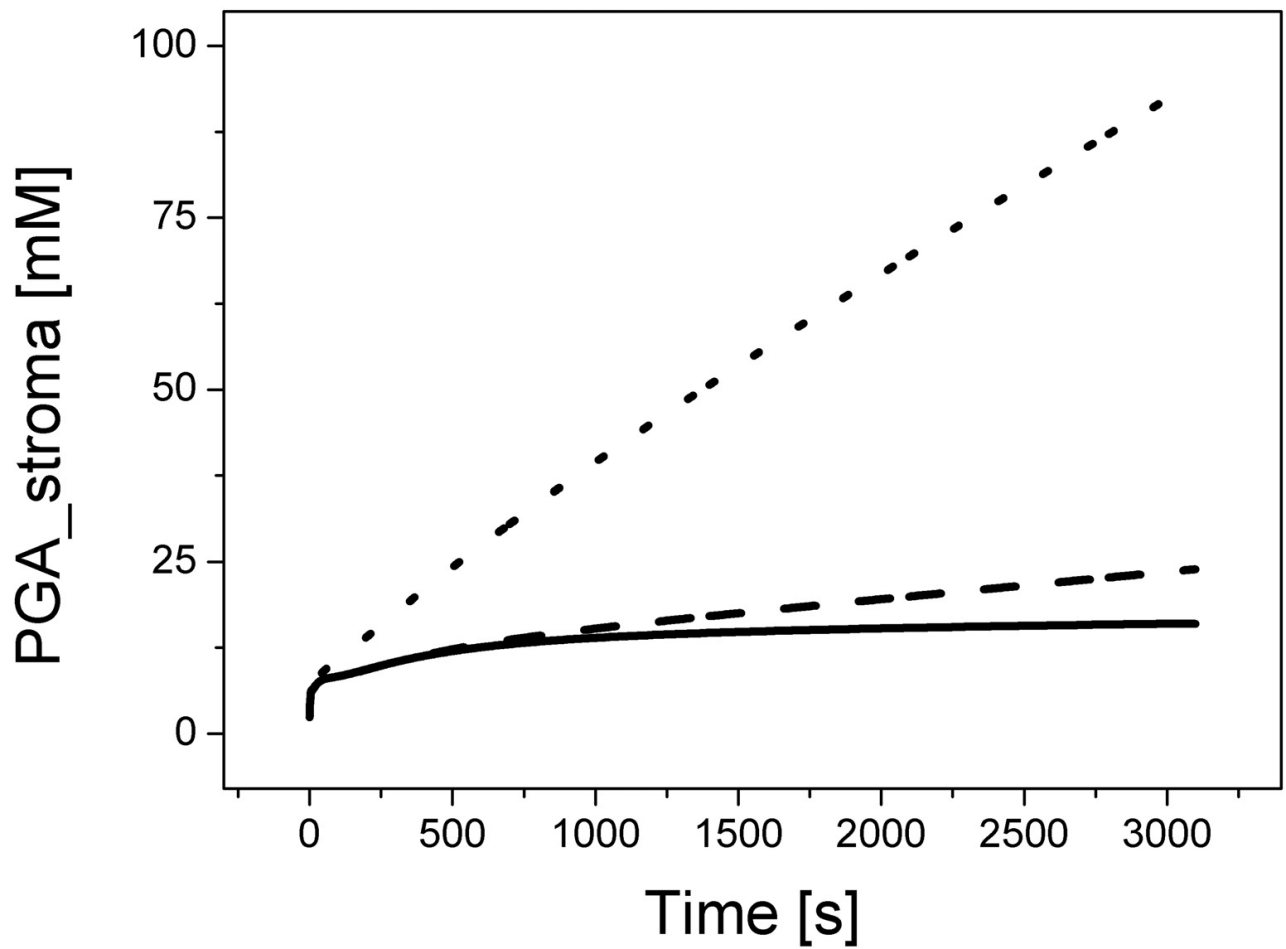
**Original and improved approaches of PGA sink implementation based on the Laisk model**. This analysis shows the comparison of original and modified implementation of the PGA sink based on the rate equation V_PG_Aout from the Laisk model [19]. **Dotted line **represents the original approach employed in the Laisk model. **Dashed line **indicates the response of the system due to 100-times increase of the stromal PGA. **Solid line **shows the extension of the equation system by adding the sink reaction (PGA_stroma _→ PGA_cytosol _→ Sink; first reaction is described by the rate equation V_PG_Aout) which was necessary to stabilize the stromal PGA due to complexity and reversibility of the rate equation V_PGAout _[19]. The initial concentrations and conditions were the same for all simulations.

The approach employed in the Laisk model has its problems as well. At first, the stromal PGA is gradually accumulated without any inhibition effect in the rate of translocator, see Figure 6. This fact contradicts what was affirmed before [19]. Moreover, the export of PGA, based on the equation for V_PG_Aout [19], does not depend, due its complexity and reversibility, on the value of maximum rate of the reaction Vm_11 _(data not shown) even if this parameter is part of the equation (see above). On the other hand, the original equation V_PG_Aout is sensitive to the changes in metabolite concentrations. For instance, if the concentration of cytosolic phosphate is increased 100-times, in comparison to stromal phosphate, the stromal PGA is decreased 4.8-times but still without any sign of saturation as shown in Figure 6. The problem with PGA accumulation in the stroma can be partially solved by adding another reaction: PGA_stroma _→ PGA_cytosol _→ Sink. This solution stabilizes the stromal PGA and defines the expected saturation, see Figure 6.

Besides the approach employed in the Laisk model for the phosphate translocator, one can find another and simple one, introduced in the Pettersson model [2] and adopted in the next model generation [3,21]. In this approach, the phosphate translocator is encoded in the Michaelis-Menten kinetics and regulated, besides the PGA, by concentration of internal/external phosphate, DHAP and GAP. We note that, in comparison to Laisk model [19], the reaction PGA_cytosol _→ Sink is redundant in this case but was employed anyway [3]. Since this approach can be used only for chloroplast, we also tested a simplified version for cyanobacteria described with the simplest Michaelis-Menten kinetics, regulated only by concentration of PGA and estimated k_M_. To have an unregulated implementation of the PGA sink to compare with, we employed also irreversible kinetics using the mass action law without modifiers. Since we were interested in how the changes within the Calvin-Benson can influence associated pathways of carbon metabolism and the efficiency of the cycle itself, we have focused our analysis on the content of metabolites within the Calvin-Benson cycle as well as on the accumulation of key products, i.e., starch and PGA sink after 3 hours in the steady state conditions.

The results of our analysis summarized in the Table 1 show several interesting outcomes. At first, it is clear that the content of metabolites in the Calvin-Benson cycle depends significantly both on the changes (related to the errors in this case) within the cycle itself, as discussed in the section above, but also on the level of regulation of PGA sink (Table 1). A similar pattern can be observed also in the case of starch synthesis. More strikingly, the steady state production of PGA depends only slightly on how/if it is regulated or if any changes occurred in equilibrium within the Calvin-Benson cycle. Finally, if we compare the efficiency of the implemented approaches, which can be measured by total mass production, unregulated approach represented by mass action kinetics is less efficient in comparison to two regulated approaches. This suggests that "export" of metabolites from the Calvin-Benson cycle is, as expected, regulated also in cyanobacteria, which do not have the phosphate translocator. However, there are no significant differences between more complex regulation, employed in Poolman and Zhu models, or here introduced simplified approach for PGA sink, see Table 1. Taken together, the employed approach for PGA sink has cardinal influence on the Calvin-Benson cycle and starch synthesis but not for the export of PGA itself.

**Table 1:** Analysis of Zhu model, versions from Matlab and Appendix A, in dependence of employed implementation of PGA sink in the model

	PGA sink	Starch	C-B pool	Total mass
**A model, MA**	82.3 (89.5)	93.3 (61.8)	29.9 (16.8)	81.6 (86.2)
**A model, MM**	96.4 (104.1)	**102.5 (83.1)**	**162.4 (51.7)**	98.4 (101.7)
**A model, MMcb**	99.3 (103.8)	101.2 (85)	74.3 (63.1)	98.8 (101.8)
**M model, MA**	87.3 (95.4)	72.8 (31.7)	17.3 (15.4)	84.9 (90.0)
**M model, MM**	100.0 (106.1)	100.0 (69.2)	100.0 (55.0)	100.0 (102.8)
**M model, MMcb**	**101.4 (105)**	98.2 (74.7)	59.6 (73.5)	100.2 (102.5)

The results show several approaches in the 3 - phosphoglycerate sink implementation and its consequence on the system efficiency (total mass production), regulation and content of the pool of metabolites as well as the changes within the Calvin-Benson after 3 hours of simulation (from dynamic changes to steady state conditions). The numbers indicates percentage changes in comparison to corrected Zhu model (100%), Matlab version. The response of system to the changes has been tested by using the original Zhu model (values in the brackets) which has significantly different equilibrium in comparison to the corrected model versions. Meaning of particular abbreviations: **C-B pool **- metabolic pool of the Calvin-Benson cycle, **A **- Appendix A model version, **M **- Matlab model version, **MA **- Mass action law kinetics, **MM **-Michaelis-Menten kinetics (phosphate translocator), **cb **- designed for cyanobacteria. The bold font indicates the highest value.

### Minor sinks for adjacent pathways, photorespiration and system efficiency

The Calvin-Benson cycle in cyanobacteria is directly linked to other parts of the carbon metabolism. The question therefore arises if all adjacent metabolic pathways have to be considered in the kinetic model. An associated problem to solve is if it is the number of sinks itself (Figure 1) or just the total flux out of the Calvin-Benson cycle which has an impact on the efficiency of the mass production. Finally, even if photorespiration is considered only in the minority of models [3,17], its flux out of the system is considered, usually in the phosphate translocator reactions, otherwise the Calvin-Benson cycle cannot reach the steady state due to infinite grow of PGA concentration (data not shown). We therefore aimed our focus also in this direction.

The starting point for our analysis was the reconstruction of the metabolic network for cyanobacteria *Synechocystis *sp. PCC 6803, adjusted for highest efficiency of the mass production [5]. We have taken the model A (corrected Zhu model - version from Appendix A; additional file 1), particularly the modified subversion for cyanobacteria (sinks only for PGA and F6P) and used this model as a template for our further modeling efforts.

### Photorespiration not directly implemented but considered within PGA sink

As previously mentioned, photorespiration does not have to be considered in the model but its flux out of the system cannot be neglected. Otherwise, the Calvin-Benson cycle cannot reach the steady state due to an accumulation of PGA. In order to analyze the consequences of this approach, together with analysis of the minor sinks, we have developed models C1 and C2. Models C were "extended" by PGCA (phosphoglycolate) sink (photorespiration) in a way which was employed in the majority of models of the Calvin-Benson cycle i.e., indirectly within the phosphate translocator. In our case, the PGCA sink was considered as a part of the PGA sink with the basic flux 4.2% of the RuBP (ribulose 1,5-biphosphate) synthesis flux [5] multiplied by a stoichiometric factor 1.66 (RuBP has 5 carbons, PGA has 3 carbons).

Both models (C1 and C2) were adapted for the same RuBP synthesis flux in the steady state (Δ flux = 5 × 10^-6^) and flux through F6P sinks, 2.4% [5] of the RuBP (ribulose 1,5-biphosphate) synthesis flux in the steady state within the Calvin-Benson cycle. The key difference between models C1 and C2 is that in the case of the model C1 (additional file 3), sinks for DHAP, E4P and Ri5P were not encoded in the model but their basic fluxes were multiplied by a stoichiometric factor q (e.g., for Ri5P, q = 1.66), summed and added to the basic flux of the PGA sink which simulates the other sinks within the PGA sink (the same approach was used for PGCA). The basic fluxes for Ri5P, DHAP and E4P sinks are 0.7%, 0.32% and 0.31% of the RuBP synthesis flux (Henning Knoop, personal communication), respectively. On the other hand, model C2 (additional file 4) was extended by sinks for DHAP, E4P and Ri5P with basic fluxes according the above mentioned proportions (Henning Knoop, personal communication).

In the case of PGA sink, the maximal and applied stable flux (value after 3 hours) was found to be equal to 18.2% in the case of C1 model (after subtraction of other sinks, see above) and 19.3% (C2 model) instead of proposed theoretical 21.7% of the RuBP synthesis flux [5]. We note that the same flux through the PGA might be achieved without disbalancing the RuBP synthesis flux in both C models by using different set of parameters for each model but the parameters may be out of the physiological range. The initial concentrations for both C models (as well as for D models) were taken from the original Zhu model which has significantly different equilibrium in comparison to the corrected version and the system was set out of the steady state.

**Figure 7:**
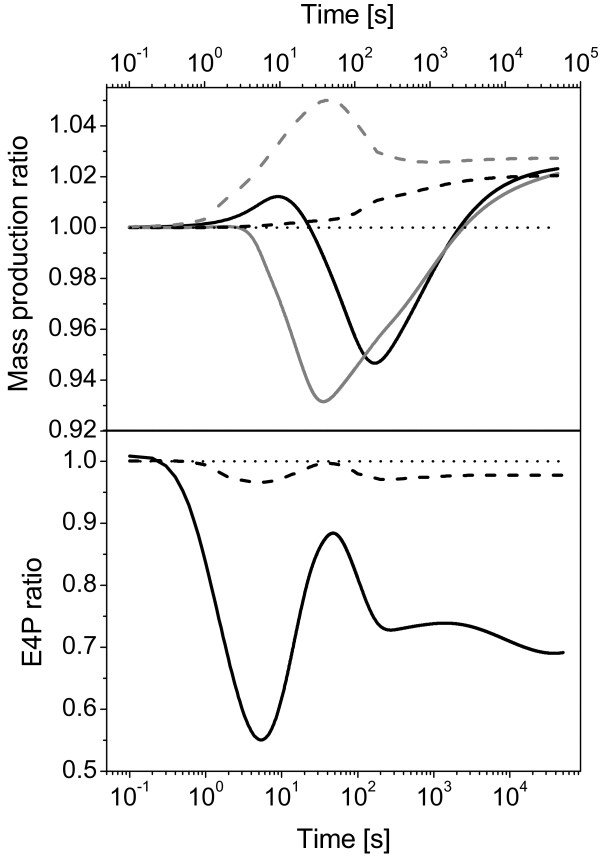
**Analysis of the system efficiency in constant steady state total flux out of the Calvin-Benson cycle and dependent on whether the minor sinks are or considered in the model or not**. Corrected Zhu model, modified for description of cyanobacteria, was used as a template for this analysis. Model **C1**: the minor sinks for DHAP, E4P, Ri5P and PGCA were implemented into the model indirectly within the PGA sink. Model **C2**: the minor sinks for DHAP, E4P, Ri5P and PGCA were encoded into the model directly (reactions described by rate equations). Model **D1**: the minor sinks for DHAP, E4P and Ri5P were implemented into the model indirectly within the PGA sink, PGCA sink is implemented as a first step of photorespiration (O_2 _fixation). **D2 **model: the minor sinks for DHAP, E4P and Ri5P were encoded into the model directly, PGCA sink is implemented as a first step of photorespiration. The basic fluxes for Ri5P, DHAP, E4P, PGA and PGCA were estimated on the basis of flux balance analysis of the cyanobacterial metabolic network [5], (Henning Knoop, personal communication). **Solid black line **represents the ratio in the mass production and E4P for models C (C1/C2); **dashed black line **represents the ratio in the mass production and E4P for models D (D1/D2); **solid gray line **shows ratio in the sinks production for models C and **dashed gray line **indicates the ratio in the sinks production for models D.

The comparison of results based on C models is striking. Having in mind that the only change between the models C1 and C2 was redistribution of the relatively small flux (1.33% of RuBP synthesis) and slight difference in the PGA sink (1.1%, values after 3 hours of the simulation), the ratios of both the total mass production and sum of sinks show dynamic changes for several hours of the simulation, see Figure 7. One can even observe a difference around 45% or even an oscillation in the ratio curve for particular metabolite (Figure 7) which, however, does occur for separate time-series data of E4P (data not shown). Finally, the presented difference in the mass production and E4P was a response to dynamic changes within the models, changes which are in certain extent inevitable even in the controlled environment.

### Photorespiration implemented directly as O_2 _fixation

Models D, in contrast to models C, implement the photorespiration directly as O_2 _fixation. At first, we have employed for the description of O_2 _fixation rate a slightly modified equation applied in the Zhu model [3], based on previous experimental and theoretical findings [30-32] (for details see [3]):

$$\frac{\text{RuBP}\times \frac{\text{Vo}\times {\text{O}}_{2}}{{\text{O}}_{2}+\text{Ko}\times \left(1+\frac{\text{C}{\text{O}}_{2}}{\text{Kc}}\right)}}{\text{RuBP}+\text{Kr}\times \left(1+\frac{\text{PGA}}{\text{KI}11}+\frac{\text{FBP}}{\text{KI}12}+\frac{\text{SBP}}{\text{KI}13}+\frac{\text{Pi}}{\text{KI}14}+\frac{\text{NADPH}}{\text{KI}15}\right)}$$

However, our simulations showed that it is not necessary to use two separated complex equations for the carboxylase and oxygenase (see the equation above) activity of RuBisCO as it was done by Zhu and coworkers [3]. It is possible to encode only one equation (the one for the carboxylase activity) and the reaction for CO_2 _fixation can be extended by additional product PGCA: RuBP + CO_2 _→ A × PGA + B × PGCA; in our case A = 1.916 and B = 0.084 (i.e., 4.2%, [5]). The differences between these two approaches are negligible both in and out of the steady state (data not shown). Moreover, our approach is easier for adjusting the level of photorespiration according our expectations and tested hypotheses and it was therefore employed in D models (see below).

Both models (D1 and D2) were again adapted for the same RuBP synthesis flux in the steady state (Δ flux = 3 × 10^-4^). The maximal and applied stable flux for PGA sink (value after 3 hours) was found to be equal to 17.1% in the case of D1 model (after subtraction of other sinks, see above) and 18.2% (D2 model) which shows slightly lower efficiency in comparison to C models.

The results based on D1 model (additional file 5) and D2 model (additional file 6) show totally different picture of what is going on during the response to perturbation until the steady state is reached, above all in the range of seconds - thousands of seconds, in comparison to C models, see Figure 7. If a model does not consider photorespiration described as oxygenase activity of RuBisCO, the difference in the export efficiency is reaching 10% (Figure 7, difference between gray lines). Furthermore, even if the time series data of particular metabolite shows the same qualitative pattern, see an example of E4P oscillation in Figure 7, the quantitative behavior demonstrates the differences in tenths of percentage. It is apparent that the content of metabolites within the Calvin-Benson cycle is less sensitive to changes induced by minor sinks implementation (Figure 7) if the photorespiration is implemented as oxygenase activity of RuBisCO.

**Figure 8:**
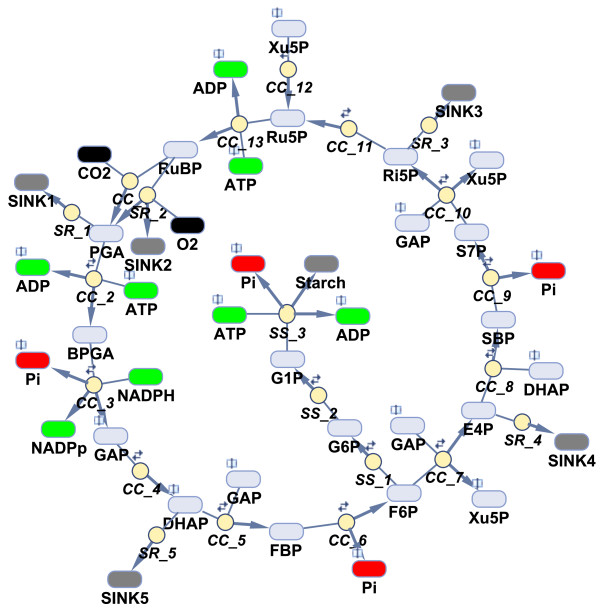
**Scheme of the model describing the cyanobacteria**. The reaction modifiers as well as the exact stoichiometry are not shown to keep the presentation simple. The abbreviations used are standard and therefore not explained [3]. Model description: The employed model includes 13 reactions for the Calvin-Benson cycle (CC_1-13), 3 reactions for starch synthesis (SS_1-3) and 5 sinks (SR_1-5); all of them entered in the form of Michaelis-Menten kinetics. The initial concentrations (except hexoses and pentoses) and kinetic parameters (except k_E7 _and k_M103_) were used as proposed by [3]. In contrast to the Zhu model, all hexoses and pentoses, as well as GAP and DHAP, are encoded separately; initial concentrations for these metabolites were adopted from [20]. The model has one compartment and assumes fixed CO_2_, O_2 _and Pi concentrations. Notes: book symbol indicates that the metabolite participates in more than one reaction; small forward/backward arrows above the circles indicate the reversibility of reactions; the products of reactions are highlighted by bold arrows.

Taken together, the comparisons indicate that it really matters how the photorespiration is incorporated into the model. Therefore, the majority of models, above all those models employing Michaelis-Menten kinetics (e.g., Pettersson model [2] or Laisk model [19]) which was also employed in our models, are not suitable for simulating the dynamic responses (e.g., change from high to low CO_2 _level) focused on analyzing the changes in metabolite content of the Calvin-Benson cycle (compare E4P ratios, Figure 7) or on mass production (Figure 7). On the other hand, the Zhu model can be employed for such analysis or modeling the photorespiration if the aforementioned corrections of the Calvin-Benson cycle (see the section *Analysis of the Zhu model and problems occurring in kinetic modeling of the Calvin-Benson cycle*) are applied. Finally, one can also conclude that the minor sinks, even with small relative fluxes, should be considered in the models of the Calvin-Benson cycle for analyses out of the steady state. The scheme of the model D2, which we recommend for next modeling efforts, is shown in Figure 8.

## Conclusions

Due to its long history of modelling, the most studied plant metabolic system, the Calvin-Benson cycle, provides a portfolio of kinetic models that can be used as a basis for further studies. What our present work reveals is that it is not possible to adapt existing and well-established models without significant modifications or corrections. We have shown that providing the basis for a look back at this history is important for further development in this field and essential to avoid the propagation of errors into new models generation.

In the present work we analyze two newer models, which are readily available and usable (the Poolman model and the Zhu model), as well as two fundamental models, referred to as the Hahn model and Pettersson model. Our study reveals that it is not possible to reproduce the simulations based on these fundamental models, probably due different (and unknown) computational methods employed in the original works [2,15]. Furthermore, in the case of two newer models, we suggest corrections for these models and strongly recommend the use of two modeling tools, in our case Copasi and Matlab, as a standard approach. This approach significantly reduces the risk of systematic errors caused, e.g. by a lack of dimensional analysis.

We also discuss possible approaches for how the 3-phosphoglycerate sink should be implemented in the model. We analyze it with respect to system efficiency, regulation and content of the pool of metabolites in the inner cycle. We further show that photorespiration or at least its first step (O_2 _fixation) has to be implemented in the model if this model is aimed for analyses out of the steady state - we suggest a simplified modification of the model, particularly of chemical equation for CO_2 _fixation without changing the rate equation or kinetic parameters. Finally, the minor adjacent pathways of the carbon metabolism have been analyzed and we show that they cannot be neglected and should be considered in the model.

Our study shows that modeling photosynthesis, in combination with a kinetic model and FBA together, constrains the model and we get more accurate results and better predictions. Further combinations of methods, for instance with non-steady state metabolic control analysis in the case of detailed focus on dynamic response, can provide even more information about the properties of the model and above all about the biological system. This approach is a promising avenue for further research.

## Competing interests

The authors declare that they have no competing interests.

## Authors' contributions

Ideas and concepts were jointly discussed among all authors. The manuscript was written by JJ and OW contributed to the writing of the final manuscript. All authors read and approved the final manuscript.

## Supplementary Material

Additional file 1 **corrected model of the Calvin-Benson cycle and starch synthesis based on the Zhu model_Appendix A version**. **model A**, SBML L2V4; model does not consider the photorespiratory pathway Click here for file

Additional file 2 **corrected model of the Calvin-Benson cycle and starch synthesis based on the Zhu model_Matlab version**. **model B**, SBML L2V4; model does not consider the photorespiratory pathway Click here for file

Additional file 3 **model_based on the corrected Zhu model_ Appendix A version**. **model C1**, SBML L2V4; modified model A for the purpose of comparison with model C2 -analysis of minor sinks and mass production efficiency; model does not consider the photorespiratory pathway but PGCA sink is considered within the PGA sink Click here for file

Additional file 4 **minor sinks model_based on the corrected Zhu model_ Appendix A version**. **model C2**, SBML L2V4; model does not consider the photorespiratory pathway but PGCA sink is considered within PGA sink, minor sinks for DHAP, E4P and Ri5P are implemented Click here for file

Additional file 5 **model_based on the corrected Zhu model_ Appendix A version**. **model D1**, SBML L2V4; modified model A for the purpose of comparison with model D2 -analysis of sinks and mass production efficiency; model does consider the first step of the photorespiratory pathway (O_2 _fixation) Click here for file

Additional file 6 **minor sinks model_based on the corrected Zhu model_ Appendix A version**. **model D2**, SBML L2V4; model does consider the first step of the photorespiratory pathway (O_2 _fixation), the minor sinks for DHAP, E4P and Ri5P are implemented Click here for file

## References

[B1] Bassham JA Krause GH Free energy changes and metabolic regulation in steady state photosynthetic carbon reduction Biochim Biophys Acta 1969 189 207 221 10.1016/0005-2728(69)90048-6 5350447

[B2] Pettersson G Ryde-Pettersson U A mathematical model of the Calvin photosynthesis cycle Eur J Biochem 1988 175 661 672 10.1111/j.1432-1033.1988.tb14242.x 3137030

[B3] Zhu XG de Sturler E Long SP Optimizing the distribution of resources between enzymes of carbon metabolism can dramatically increase photosynthetic rate: a numerical simulation using an evolutionary algorithm Plant Physiol 2007 145 513 526 10.1104/pp.107.103713 17720759 PMC2048738

[B4] Boyle NR Morgan JA Flux balance analysis of primary metabolism in *Chlamydomonas reinhardtii* BMC Syst Biol 2009 3 4 19128495 10.1186/1752-0509-3-4 PMC2628641

[B5] Knoop H Zilliges Y Lockau W Steuer R The Metabolic Network of Synechocystis sp. PCC 6803: Systemic Properties of Autotrophic Growth Plant Physiology 2010 154 410 422 10.1104/pp.110.157198 20616194 PMC2938163

[B6] Montagud A Navarro E de Cordoba PF Urchueguía JF Patil KR Reconstruction and analysis of genome-scale metabolic model of a photosynthetic bacterium BMC Syst Biol 2010 4 156 10.1186/1752-0509-4-156 21083885 PMC3009638

[B7] Fleming RMT Thiele I Provan G Nasheuer HP Integrated stoichiometric, thermodynamic and kinetic modelling of steady state metabolism JTB 2010 264 683 692 10.1016/j.jtbi.2010.02.044 PMC2868105 20230840

[B8] Tenazinha N Vinga S A Survey on Methods for Modeling and Analyzing Integrated Biological Networks TCBB 2011 8 943 958 10.1109/TCBB.2010.117 21116043

[B9] Laisk A Eichelmann H Oja V C3 photosynthesis in silico Photosynth Res 2006 90 45 66 17131095 10.1007/s11120-006-9109-1

[B10] Safranek D Cerveny J Klement M Pospisilova J Brim L Lazar D Nedbal L E-photosynthesis: Web-based platform for modeling of complex photosynthetic processes BioSystems 2011 103 115 124 10.1016/j.biosystems.2010.10.013 21073914

[B11] Zhu X Alba R de Sturler E A simple model of the Calvin cycle has only one physiologically feasible steady state under the same external conditions Nonlinear Anal-Real 2009 3 1490 1499

[B12] Grimbs S Arnold A Koseska A Kurths J Selbig J Nikoloski Z Spatiotemporal dynamics of the Calvin cycle: Multistationarity and symmetry breaking instabilities BioSystems 2011 103 212 223 10.1016/j.biosystems.2010.10.015 21075168

[B13] Hoops S Sahle S Gauges R Lee C Pahle J Simus N Singhal M Xu L Mendes P Kummer U COPASI - a COmplex PAthway SImulator Bioinformatics 2006 22 3067 74 10.1093/bioinformatics/btl485 17032683

[B14] Lazar D Kana R Klinkovsky T Naus J Experimental and theoretical study on high temperature induced changes in chlorophyll *a *fluorescence oscillations in barley leaves upon 2% CO2 Photosynthetica 2005 3 13 27

[B15] Hahn BD A mathematical model of leaf carbon metabolism Annals of Botany 1984 54 325 339

[B16] Hahn BD A Mathematical Model of the Calvin Cycle: Analysis of the Steady State Annals of Botany 1986 57 639 653

[B17] Hahn BD A mathematical model of photorespiration and photosynthesis Annals of Botany 1987 60 157 169

[B18] Woodrow IE Control of the rate of photosynthetic carbon dioxide fixation Biochim Biophy Acta 1986 851 181 192 10.1016/0005-2728(86)90124-6

[B19] Laisk A Eichelmann H Oja V Eatherall A Walker DA A mathematical model of the carbon metabolism in photosynthesis. Difficulties in explaining oscillations by fructose 2,6-bisphosphate regulation Proc R Soc Lond B Biol Sci 1989 237 389 415 10.1098/rspb.1989.0057

[B20] Woodrow IE Mott KA Modeling C3 photosynthesis--a sensitivity analysis of the photosynthetic carbon reduction cycle Planta 1993 191 421 432

[B21] Poolman MG Assmus HE Fell DA Applications of metabolic modelling to plant metabolism J Exp Bot 2004 55 1177 1186 10.1093/jxb/erh090 15073223

[B22] Poolman MG Computer modeling applied to the Calvin cycle: PhD thesis 1999 Oxford Brookes University

[B23] Kallas T Castenholz RW Internal pH and ATP-ADP pools in the cyanobacterium Synechococcus sp. During exposure to growth-inhibiting low pH Journal of Bacterology 1982 149 229 236 10.1128/jb.149.1.229-236.1982 PMC216614 6798019

[B24] Portis AR Chon CJ Mosbach A Heldt HW Fructose- and sedoheptulosebisphosphatase. The sites of a possible control of CO2 fixation by light-dependent changes of the stromal Mg 2+ concentration Biochim Biophys Acta 1977 461 313 325 10.1016/0005-2728(77)90181-5 19060

[B25] Stitt M Wirtz W Heldt HW Metabolite levels during induction in the chloroplast and extrachloroplast compartments of spinach protoplasts Biochim Biophys Acta 1980 593 85 102 10.1016/0005-2728(80)90010-9 7426648

[B26] Sprenger GA Schorken U Sprenger G Sahm H Transketolase A of Escherichia coli K12. Purification and properties of the enzyme from recombinant strains Eur J Biochem 1995 230 525 532 10.1111/j.1432-1033.1995.0525h.x 7607225

[B27] Woodrow IE Mott KA Rate limitation of non-steady-state photosynthesis by ribulose-1,5-bisphosphate carboxylase in spinach Aust J Plant Physiol 1989 16 487 500 10.1071/PP9890487

[B28] Kana R Kotabova E Prasil O Acceleration of plastoquinone pool reduction by alternative pathways precedes a decrease in photosynthetic CO2 assimilation in preheated barley leaves Physiol Plant 2008 133 794 806 10.1111/j.1399-3054.2008.01094.x 18494737

[B29] Albe KR Partial purification and kinetic characterization of transaldolase from *Dictyostelium discoideum* Exp Mycol 1991 15 255 62 10.1016/0147-5975(91)90027-B

[B30] Badger MR Lorimer G Interaction of sugar phosphate with the catalytic site of RuBP-carboxylase Biochemistry 1981 20 2219 2225 10.1021/bi00511a023 7236594

[B31] Farquhar GD Models describing the kinetics of ribulose biphosphate carboxylase-oxygenase Arch Biochem Biophys 1979 193 456 468 10.1016/0003-9861(79)90052-3 464606

[B32] von Caemmerer S Biochemical Models of Leaf Photosynthesis 2000 Victoria, Australia 1 28 Techniques in Plant Sciences Series. CSIRO Publishing

